# Feeling Virtually Present Makes Me Happier: The Influence of Immersion, Sense of Presence, and Video Contents on Positive Emotion Induction

**DOI:** 10.1089/cyber.2022.0245

**Published:** 2023-04-14

**Authors:** Katarina Pavic, Laurence Chaby, Thierry Gricourt, Dorine Vergilino-Perez

**Affiliations:** ^1^ Université Paris Cité, Vision Action Cognition (VAC), Boulogne-Billancourt, France.; ^2^ Sorbonne Université, CNRS, Institut des Systèmes Intelligents et de Robotique (ISIR), Paris, France.; ^3^ Research and Development Department, SocialDream, Bourg-de-Péage, France.; ^4^ Université Paris Cité, UFR de Psychologie, Boulogne-Billancourt, France.

**Keywords:** Virtual Reality, positive emotion, sense of presence, physiological data, positive technology

## Abstract

Immersive technologies, such as Virtual Reality (VR), have great potential for enhancing users' emotions and wellbeing. However, how immersion, Virtual Environment contents, and sense of presence (SoP) influence emotional responses remains to be clarified to efficiently foster positive emotions. Consequently, a total of 26 participants (16 women, 10 men, 22.73 ± 2.69 years old) were exposed to 360-degree videos of natural and social contents on both a highly immersive Head-Mounted Display and a low immersive computer screen. Subjective emotional responses and SoP were assessed after each video using self-reports, while a wearable wristband collected continuously electrodermal activity and heart rate to record physiological emotional responses. Findings supported the added value of immersion, as more positive emotions and greater subjective arousal were reported after viewing the videos in the highly immersive setting, regardless of the video contents. In addition to usually employed natural contents, the findings also provide initial evidence for the effectiveness of social contents in eliciting positive emotions. Finally, structural equation models shed light on the indirect effect of immersion, through spatial and spatial SoP on subjective arousal. Overall, these are encouraging results about the effectiveness of VR for fostering positive emotions. Future studies should further investigate the influence of user characteristics on VR experiences to foster efficiently positive emotions among a broad range of potential users.

## Introduction

Being happy today can have unexpected benefits for tomorrow. For instance, happiness has long-term benefits on people's quality of life,^1^ health,^2^ and could lead to wellbeing and fulfillment.^3^ Thus, it appears relevant to investigate how happiness, and more broadly positive emotions, can be fostered. One way of fostering positive emotions lies in the “positive technologies” framework, which suggests that technologies could enhance users' emotions, experiences, and wellbeing.^4–6^ Cited within this framework, Virtual reality (VR) has great potential, as it enables users to have experiences in safe and controlled environments,^6,7^ and can trigger a change in users' emotions.^8,9^ However, as several socioeconomic barriers have been identified regarding the widespread use of VR to improve wellbeing,^10^ it is necessary to investigate which characteristics of VR are crucial to efficiently foster positive emotions.

First, the level of immersion required to foster positive emotions must be clarified. Immersion usually refers to the objective ability of a technology to deliver multisensory stimulation equivalent to real life.^11^ In this context, it is possible to foster positive emotions by inducing temporary emotional states,^12,13^ usually through the use of emotionally arousing pictures,^14^ videos,^15^ music,^16^ or sentences.^17^ However, mixed results have been reported about the added value of high levels of immersion compared with usual low-immersive screen presentations.^18–21^ Since most studies have relied solely on self-report measures, combining self-report and physiological measures may help to gain the broader picture.^22–24^

Second, it is also important to investigate the content of Virtual Environments (VEs) used to evoke positive emotions. This issue had been largely ignored, as previous studies have relied primarily on natural VE contents because of their well-known relaxing properties.^25–27^ A recent study highlighted that, when participants were asked to record personalized 360-degree videos, most of them included the presence of at least one person in their recordings.^28^ In addition, social contents are reported to induce higher levels of positive emotions and arousal than nonsocial contents,^29,30^ yet this remains to be further tested when using immersive technologies. Overall, it is relevant to evaluate the effectiveness of VEs containing social features (e.g., being surrounded by people) to evoke positive emotions.

Finally, the role of the “Sense of Presence” (SoP) needs to be addressed. SoP is a key characteristic of VR and refers to the feeling of “being there physically” (i.e., spatial presence^31^), to which can be added the feeling of “being there with others” (i.e., social presence^32^). Although SoP and immersion are interconnected, it is not entirely clear how each one contributes to positive emotion induction. It would be helpful to clarify whether the induction of positive emotion is solely dependent on the immersive properties of a technology, or whether SoP mediates this relationship. The mediating role of SoP is worth exploring, as it is known that higher levels of immersion lead to greater SoP,^18,33,34^ and that higher levels of SoP can lead to greater arousal.^19,24,35^

Thus, the present study aims at investigating the level of immersion and VE contents required to induce positive emotions by employing both “subjective” (i.e., self-report), and “objective” (i.e., physiological) measures of induced emotions. It was hypothesized that higher levels of immersion would be more efficient in conveying positive emotions and would lead to greater arousal compared with lower levels of immersion. We expected the effect of immersion to be even greater when combined to social VE contents compared with natural ones. Moreover, the present study adds to the literature by investigating the mediator roles of SoP between immersion and emotional arousal.

## Methods

### Participants

Based on a meta-analysis about the effectiveness of videos for inducing emotions,^36^ a power analysis aimed at detecting medium-size effects (*f* = 0.29, α = 0.05, β = 0.80) was conducted in G*Power 3.0^37^ for a within-subject repeated measures analysis of variance (ANOVA), suggesting a minimal sample of 25 participants. Initially, 28 healthy adults were recruited. Outlier detection led to the removal of two participants based on their physiological data. Thus, the final sample consisted of 26 participants (16 women, 10 men, 22.73 ± 2.69 years old). Participants reported few-to-no depressive symptoms on the Hospital Anxiety and Depression Scale (HADS^38^). The research was approved by the Ethics Committee of Université Paris Cité (IRB No. 00012021-61). All participants provided written consent and received a compensation of 15 euros.

### Materials

The stimuli consisted of nine 360-degree videos, 1 neutral and 8 videos, designed to induce positive emotions.

The neutral “control” video was created using Unity 2021.1.0 and consisted of an empty room with an open door and shapes on the walls. The other videos were created with a GoPro Fusion 360 camera and consisted of four “nature” videos (i.e., vegetation or aquatic features), and four “social” videos (i.e., seeing smiling people on a stroll or at a concert). All the videos had a 4K resolution and sounds in accordance with the context. Motions relied on “teleportation,” which consists in changing the viewpoint with visual “jumps” from one point to another.^39^ The control video consisted of two teleportation motions launched by the experimenter: one toward the door, and a second one back to the center of the room. The nature and social videos consisted of six teleportation motions that occurred automatically every 20 seconds. All the videos began with a black waiting screen lasting 10 seconds, followed by a control, natural, or social video content unfolding for 2 minutes. The control video content had an additional training phase not included in the 2 minutes.

Two technologies were employed to compare levels of immersion: a highly immersive Head-Mounted Display (HMD) (Samsung HMD Odyssey+, 1,440 × 1,600 pixels resolution) and a less-immersive computer screen (25-inch Iiyama screen, 1,920 × 1,080 pixels resolution). The videos were explored by head movements under the HMD, or mouse movements on the screen.

Emotional responses were assessed with both self-report and physiological measures. Valence and arousal were self-reported on the Self-Assessment Manikin^40^. In addition, eight 7-point Likert scales were used to measure positive (excitement, joy, relaxation, interest) and negative (anxiety, anger, sadness, boredom) affects. Physiological responses were acquired with an Empatica E4 wristband, namely Electrodermal Activity (EDA, 4 Hz) and heart rate (HR, 1 Hz). The Spatial Presence Experience Scale^41^ (7-point Likert scales) and the Social Richness subscale of the Temple Presence Inventory^42^ (5-point Likert scales) were employed as self-report measures of spatial and social SoP.

### Procedure

**FIG. 1.**
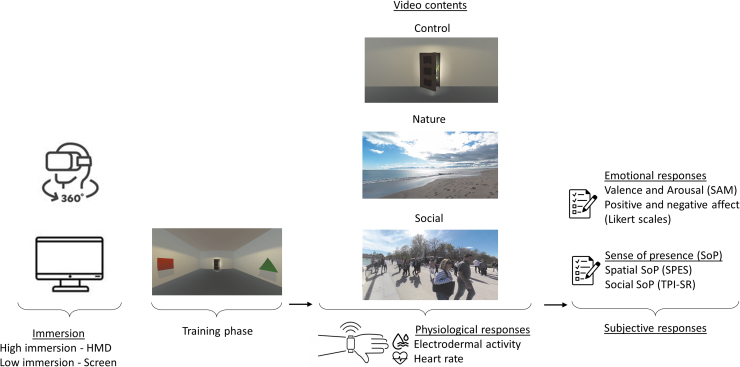
Graphical representation of the experimental procedure. All participants viewed 360-degree videos on both technologies in a counterbalanced order. HMD, Head-Mounted Display; SAM, Self-Assessed Manikin; SPES, Spatial Presence Experience Scale; TPI-SR, Temple Presence Inventory—Social Richness subscale.

On arrival, participants answered demographic questions, completed the HADS, and the wristband was placed on their nondominant hand. Each participant was exposed to 360-degree videos on both screen and HMD in a counterbalanced order. For both levels of immersion, participants followed the same procedure (Fig. 1). They started by a training phase in the control video to get used to the motions and the exploration of 360-degree videos. Then, participants were asked to relax and watch for 2 minutes the same control video as the one presented during the training phase while their physiological data were being collected. Afterward, they watched two natural and two social videos in a randomized order. Physiological data were acquired while viewing each video content, for a total duration of 2 minutes. After each video, participants reported their emotional responses and SoP on relevant scales. Once participants viewed five videos (one control, two natural, and two social contents) on one technology, they switched to the second one and viewed the remaining videos following the same procedure.

Participants were seated throughout the procedure, which lasted 1 hour.

## Results

Participants reported moderate levels of anxiety (*M* = 11.00 ± 4.10) and low levels of depression (*M* = 4.19 ± 2.94). Gender did not yield any significant effect (*p_s_* > 0.1).

For ANOVA analyses, Greenhouse–Geisser corrections were applied if sphericity assumptions were not met. For clarity's sake, uncorrected degrees of freedom are reported. When relevant, Bonferroni *post hoc*s were conducted.

### Subjective emotional responses

A two-way within-subjects ANOVA was conducted on valence ratings (two Immersive technologies: HMD vs. Screen × 3 Video contents: Control vs. Nature vs. Social). A significant main effect of Immersion emerged (*F*(1, 25) = 13.01; *p* = 0.001; $\eta_{p}^{2}$ = 0.34), meaning more positive emotions were reported in the highly immersive setting (*M_HMD_* = 6.95 ± 1.32) than in the less-immersive setting (*M_Screen_* = 6.28 ± 1.38). A main effect of Content (*F*(2, 50) = 21.62; *p* < 0.001, $\eta_{p}^{2}$ = 0.46) revealed that nature (*M* = 6.85 ± 1.06) and social (*M* = 7.28 ± 1.01) contents induced more positive emotions than the control content (*M* = 5.71 ± 1.54, *p_s_* < 0.001). No significant difference emerged between nature and social contents in terms of valence (*p* = 0.26). The Immersion × Content interaction was not significant (*F*(2, 50) = 0.50; *p* = 0.61, $\eta_{p}^{2}$ = 0.02). Comparable results were found on the Likert scales assessing positive and negative affects (Supplementary Material S1).

**FIG. 2.**
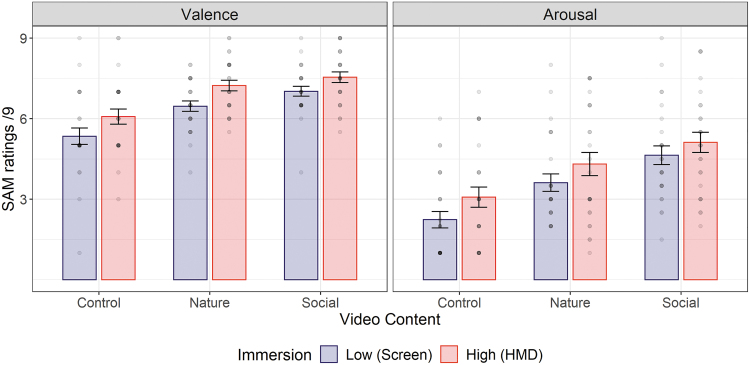
Mean valence and arousal SAM scores for each content and level of immersion. Error bars indicate standard errors of the mean. *Circles* represent individual ratings; their *brightness* indicates the number of participants giving the same rating.

A similar ANOVA carried out on arousal ratings revealed a significant main effect of Immersion (*F*(1, 25) = 5.18; *p* = 0.03, $\eta_{p}^{2}$ = 0.17), meaning greater arousal was reported in the highly immersive setting (*M_HMD_* = 4.17 ± 2.16) than in the less immersive setting (*M_Screen_* = 3.50 ± 1.92). A main effect of Content (*F*(2, 50) = 31.44; *p* < 0.001, $\eta_{p}^{2}$ = 0.56) revealed that the control content was perceived as less arousing (*M* = 2.66 ± 1.78) than the nature one (*M* = 3.96 ± 1.95, *p* < 0.001), which in turn was perceived as less arousing than social contents (*M* = 4.88 ± 1.85, *p* = 0.006). The Immersion × Content interaction failed to reach significance (*F* < 1). Mean valence and arousal ratings are illustrated on Figure 2.

### Physiological responses

Three-way within-subjects ANOVAs were conducted on EDA and HR to investigate their time-course during video watching (two Immersive technologies: HMD vs. Screen × 3 Video contents: Control vs. Nature vs. Social × 6 Time bins: 0–20, 20–40, 40–60, 60–80, 80–100 and 100–120 seconds). Physiological data collected during the training phases and outside the window of interest were not considered for statistical analyses.

**FIG. 3.**
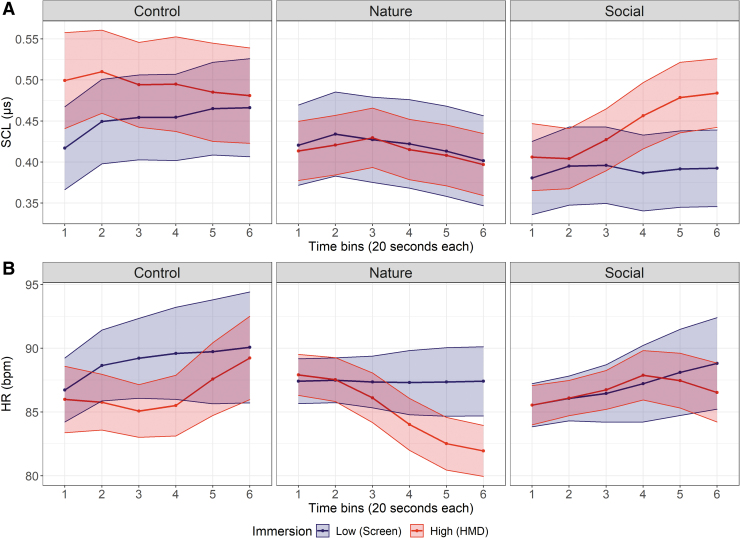
Mean time course (20-s bins) of **(A)** the range-corrected SCL and **(B)** the HR during video viewing. *Ribbons* indicate standard errors of the mean. HR, heart rate; SCL, Skin Conductance Level.

For EDA, a Continuous Decomposition Analysis was performed using LEDALAB V3.4.9 to extract the Skin Conductance Level (SCL). SCL data were range-corrected according to previous recommendations.^43,44^ Only a significant Immersion × Content × Time interaction emerged (*F*(10, 250) = 2.99; *p* = 0.05, $\eta_{p}^{2}$ = 0.11). *Post hoc* analyses only showed a tendency for SCL to increase between the beginning (*M_time bin 2_* = 0.40 ± 0.19) and the end (*M_time bin 6_* = 0.48 ± 0.21) of social video contents when watched in the highly immersive setting (*p* = 0.06, Fig. 3A). No other significant effect or interactions emerged (all *F_s_* < 1; *p_s_* > 0.1).

For HR, none of the main effect, two- and three-way interactions reached significance after applying sphericity correction (all *F*_s_ < 1; *p_s_* > 0.1), despite visible HR deceleration during natural videos in the highly immersive setting (Fig. 3B).

### Sense of presence

Two-way within-subjects ANOVAs were conducted on SoP measures (two Immersive technologies: HMD vs. Screen × 3 Video contents: Control vs. Nature vs. Social). Descriptive statistics and *post hoc* tests are reported for spatial SoP in Table 1 and for social SoP in Table 2.

**Table 1.** Mean and Standard Deviation Values of Spatial Sense of Presence (Spatial Presence Experience Scale)

Immersion	Video contents			Post hoc analyses		
	Control (*M *±* SD*)	Nature (*M *±* SD*)	Social (*M *±* SD*)	Control vs. nature	Control vs. social	Nature vs. social
Low (screen)	1.79 ± 0.85	2.55 ± 0.80	2.72 ± 0.92	***t =*** **−5.53, *p*** < **0.001**	***t =*** **−6.81, *p*** < **0.001**	*t =* −1.28, *p* = 1
High (HMD)	3.07 ± 0.98	3.41 ± 0.97	3.51 ± 01.02	*t =* −2.49, *p* = 0.22	***t =*** **−3.21, *p* = 0.03**	*t =* −0.72, *p* = 1
*Post hoc* analyses Screen vs. HMD	***t =*** **−6.81, *p*** < **0.001**	***t =*** **−4.59, *p*** < **0.001**	***t =*** **−4.18, *p* = 0.002**	**—**	**—**	**—**

Bold values indicate statistical significance (*p* < 0.05).

*Post hoc* analyses with Bonferroni corrections are reported when relevant.

HMD, Head-Mounted Display; *M*, mean; *SD*, standard deviation.

**Table 2.** Mean and Standard Deviation Values of Social Sense of Presence (Social Richness Subscale of the Temple Presence Inventory)

Immersion	Video contents			Post hoc analyses		
	Control (*M *±* SD*)	Nature (*M *±* SD*)	Social (*M *±* SD*)	Control vs. nature	Control vs. social	Nature vs. social
Low (screen)	2.19 ± 0.85	3.91 ± 0.76	4.86 ± 1.01	***t =*** **−9.06, *p* < 0.001**	***t =*** **−14.15, *p* < 0.001**	***t =*** **−5.09, *p* < 0.001**
High (HMD)	2.81 ± 0.99	4.43 ± 0.92	5.36 ± 0.78			
*Post hoc* analysis Screen vs. HMD	***t =*** **−3.44, *p* = 0.002**			—	—	—

Bold values indicate statistical significance (*p* < 0.05).

*Post hoc* analyses with Bonferroni corrections are reported when relevant.

The ANOVA conducted on spatial SoP revealed a significant main effect of Immersion (*F*(1, 25) = 32.64; *p* < 0.001, $\eta_{p}^{2}$ = 0.57), Content (*F*(2, 50) = 20.16; *p* < 0.001, $\eta_{p}^{2}$ = 0.45), and an Immersion × Content interaction (*F*(2, 50) = 5.80; *p* = 0.01, $\eta_{p}^{2}$ = 0.19). For social SoP, significant effects of Immersion (*F*(1, 25) = 11.85; *p* = 0.002, $\eta_{p}^{2}$ = 0.32), and Content (*F*(2, 50) = 102.75; *p* < 0.001, $\eta_{p}^{2}$ = 0.80) emerged. The Immersion × Content interaction failed to reach significance (*F* < 1).

### Mediation analyses

**FIG. 4.**
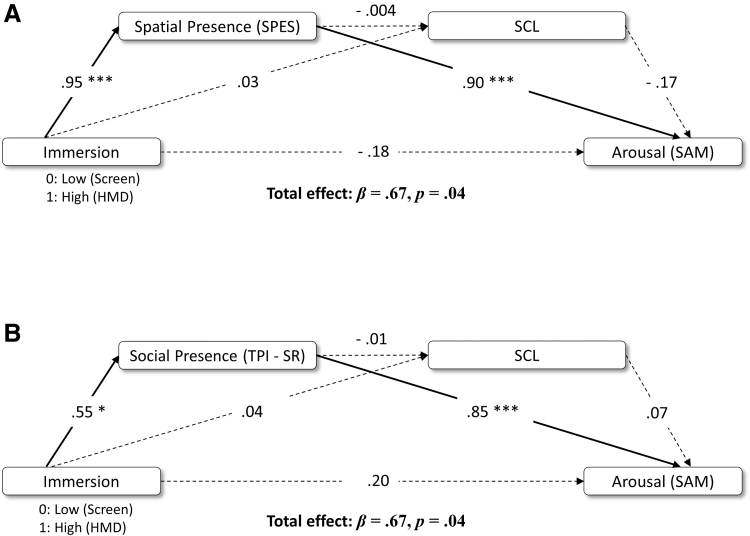
Serial Mediation Models for the association between immersion and self-reported arousal through **(A)** Spatial SoP and SCL and **(B)** Social SoP and SCL. Standardized β are reported for each path. p*<0.05, **<0.01, ***<0.001. SoP, sense of presence.

The R package “Lavaan” was used to structure and test models with 5,000 bootstrap samples. We tested two serial models, one with spatial SoP and SCL as mediators (Fig. 4A) and a second model with social SoP and SCL as mediators (Fig. 4B) of the relation between immersion and self-reported arousal. We assumed a causal chain in which immersion influenced SoP, which predicted SCL, which in turn increased self-reported arousal. Before model testing, a regression analysis confirmed that immersion predicted arousal (β = 0.67, *p* = 0.04).

The first model revealed a significant indirect effect of immersion, through spatial SoP on subjective arousal (β = 0.86, *p* < 0.001, confidence interval [CI] [0.49, 1.31]). This model did not support an indirect effect of SCL (β = −0.01, *p* = 0.88, CI [−0.1, 0.07]), nor the serial mediation, including spatial SoP and SCL as mediators (β = 0.001, *p* = 0.96, CI [−0.02, 0.03]). Similarly, the second model revealed only a significant indirect effect of immersion through social SoP on subjective arousal (β = 0.47, *p* = 0.02, CI [0.1, 0.88]). Again, the model did not support an indirect effect of SCL (β = 0.002, *p* = 0.94, CI [−0.06, 0.07]) nor the serial mediation, including social SoP and SCL as mediators (β = −0.001, *p* = 0.95, CI [−0.01, 0.01]). In summary, models demonstrate that spatial and social SoP are important mediators of the relation between immersion and subjective, but not physiological, arousal.

## Discussion

VR has great potential to enhance users' emotions and wellbeing as a positive technology.^4–6^ However, the influence of key characteristics of VR experiences on the elicitation of positive emotions remained to be addressed. Thus, the aim of this study was to understand how immersion, VE contents, and SoP contribute to positive emotion induction for successfully promoting wellbeing with VR.

First, this study examined the added value of immersion in eliciting positive emotions. As expected, higher levels of immersion (i.e., HMD presentation) elicited more positive emotions and greater arousal compared with lower levels of immersion (i.e., screen presentation). This was mostly apparent for self-reported measures, regardless of the video contents. Contrary to our expectations, immersion failed to produce similar results on physiological responses. Nevertheless, discrepant results have been reported regarding the influence of immersion on physiological responses in terms of valence and arousal.^18,20,21,44^ This emphasizes the need to further investigate which physiological indicators are relevant for reliably detecting positive emotions. Furthermore, as subjective and physiological responses are not necessarily associated,^21,25^ variations in subjective responses remain valuable indicators of users' emotional states.

Second, we have examined which VE contents are efficient in eliciting positive emotions. Indeed, there has been a lack of focus on the VE contents potentially relevant to this goal, since most studies relied on natural VEs.^45^ Our results expand previous researches by supporting the assumption that social VE contents are efficient for eliciting positive emotions in addition to natural ones. As expected, social contents led overall to greater subjective arousal, and increased physiological arousal when viewed in highly immersive settings. Specific emotional responses to social contents could be explained by distinct neural circuits activated in response to “social” and “nonsocial” emotional stimuli,^46^ and/or resulting from emotional contagion in social contexts (i.e., converging emotionally with others^47^). As the lack of diversity in VE contents was identified as one of the limitations preventing the use of VR for wellbeing,^10^ future studies should seek to further validate social and nonsocial contents.

Finally, this research aimed at exploring the links between SoP, immersion, and emotions. Our findings established both spatial and social SoP as mediators of the relationship between immersion and subjective arousal when eliciting positive emotions. Thus, rather than being solely dependent on the immersive properties of a technology, subjective emotional responses are influenced by the SoP that emerges from immersion. These results suggest that, when eliciting positive emotions with VR, it is better to focus on increasing spatial or social SoP rather than the level of immersion. It is possible to enhance SoP even with weakly immersive devices, for instance by using a larger screen size,^48^ increasing agency^49^ or creating a narrative^50^ in the VEs. Lastly, the lack of mediation between immersion, SoP, and physiological arousal is likely linked to weak physiological responses observed in our study. Using more emotionally arousing stimuli may help clarify if there are links between immersion, SoP, and physiological arousal.

### Limitations and future research

While our results provide insight into which VE contents can be used for eliciting positive emotions, it should be noted that participants were passive observers in our 360-degree videos. Future studies should investigate the benefits of interactivity as it seems even more efficient for eliciting emotions.^51,52^ Integrating interactivity may be even more relevant when employing social contents that may call for social interactions.^53^ Another limitation of our material may lie in the duration of the videos. Even though a 2-minute duration is known to be sufficient for eliciting emotions on both subjective and physiological levels,^15,30^ it is plausible that longer videos may be necessary to observe more pronounced physiological responses than in the present study.

Although we sought to clarify the aspects of VR that are important for fostering positive emotion, our findings cannot be generalized to all potential users, since only healthy young adults were recruited for the present study. Future studies should strive at gaining further evidence of VR's effectiveness for fostering positive emotions among more vulnerable users. Additionally, as we focused on how VR features influenced users' emotions, how user characteristics (e.g., age,^54,55^ or mental imagery skills^56^) influence VR experiences remains to be tested.

## Conclusions

Our findings highlight that highly immersive VR is efficient in eliciting positive emotions on self-reported emotions, and to a lesser degree, on physiological responses. For the first time, 360-degree social video contents were successfully employed to elicit positive emotions and turned out to be as efficient as natural contents. Further exploration revealed that the inductive power of VR can be explained by an indirect relationship linking immersion to SoP, which in return elicited more intense subjective emotional responses. This suggests that increasing SoP may be a valuable alternative to highly immersive devices for eliciting positive emotions. Altogether, this study highlights the methodological aspects that need to be considered to effectively foster positive emotions with VR, as well as encouraging results regarding its effectiveness.
